# Guideline-Based Early Detection of Chronic Obstructive Pulmonary Disease in Eight Danish Municipalities: The TOP-KOM Study

**DOI:** 10.1155/2017/7620397

**Published:** 2017-02-20

**Authors:** Ulla Borup Hemmingsen, Margit Stycke, Jens Dollerup, Peter Bo Poulsen

**Affiliations:** ^1^Municipality of Vordingborg, Health Secretariat, Langgade 57, 4780 Stege, Denmark; ^2^Municipality of Billund, Department of Health, Sydtoften 102, 7200 Grindsted, Denmark; ^3^Pfizer Denmark ApS, Lautrupvang 8, 2750 Ballerup, Denmark; ^4^Dmc Dollerup Medical Consultancy, Holmegårdsvej 49, 3100 Hornbæk, Denmark

## Abstract

*Background*. Early detection of chronic obstructive pulmonary disease (COPD) and prevention of disease progression are important. Only 40% of COPD cases are diagnosed in Denmark. Recommendations for early case finding have been established. This study investigates early detection of pulmonary obstruction in a Danish municipality setting.* Methods*. Eight municipalities participated. Citizens fulfilling national case finding recommendations, age ≥35 years, smokers/ex-smokers/relevant occupational exposure, and at least one respiratory symptom, were invited to spirometry. Citizens with indication of pulmonary obstruction, forced expiratory volume in one second (FEV_1_)/forced vital capacity (FVC) < 0.70, were referred to their general practitioner (GP).* Results*. 1,499 citizens were examined (53.6% male, mean age 57.2 years). 44.8% were current smokers with 57% planning for smoking cessation. The citizens recorded significant airway symptoms with dyspnea being the most important (71%). The mean FEV_1_/FVC score was 73.54 (SD 22.84). 456 citizens (30.4%) were found to have indication for pulmonary obstruction and were referred to GP for further diagnosis.* Conclusion*. Early detection in Danish municipalities proved effective finding nearly 1/3 being pulmonary obstructive. It seems to be of value to have municipalities to perform case finding together with smoking cessation as a primary intervention in COPD management.

## 1. Introduction

Chronic obstructive pulmonary disease (COPD) is characterized by airflow limitation that is not fully reversible [1, 2]. COPD is prevalent in Denmark and it is estimated that up to 430,000 (10% of the adult population) suffer from the disease [3], which will then be among the highest prevalences in Europe (OECD) [4]. Hospitalizations, medicine consumption, mortality, burden of disease, and costs of illness are high [5, 6]. A recent study has documented patients even five years before the COPD diagnosis is a burden to the society with increased direct and indirect cost compared to matched controls [7]. However, only between 150,000–170,000 patients have been diagnosed with COPD [8], calling for early diagnosis in order to counteract the accelerated fall in lung function, progression of symptoms, and development of comorbidities at a stage where the lung function is not reduced to a critical level. Spirometry is essential in early diagnosis, as symptoms are not always a reliable indicator of COPD [8].

Early detection of COPD by case finding opens possibility of early intervention and could among other things be particularly focused together with intensified smoking cessation activities. The reasons to this are that smoking is the highest risk factor for developing COPD and smoking cessation is the primary intervention that could positively modify the accelerated progression of lung function decline and complications in COPD [9]. Among patients detected and diagnosed with COPD 64% were current smokers [10] compared to 17% in the total population of daily smokers [11]. Approximately, 25% with moderate to severe COPD are still smoking [12]. It is estimated that smoking accounts for more than 80% of the COPD cases in Denmark [13].

Current and past guidelines issued by the Danish National Board of Health for the management of COPD include assessment and monitoring of the disease, reduction of risk factors to prevent progression, management of stable COPD, management of exacerbations, and case finding for prevalent comorbidities [8, 14]. For newly diagnosed and stable COPD patients, this implies initiation of smoking cessation for cigarette smokers, physical rehabilitation, and optimal pharmacotherapy for symptom relief, as well as patient education, dietary supervision, and psychosocial assistance [15–17]. The general practitioner (GP) is the coordinator in these initiatives [14]. However, patients should be found and diagnosed with COPD in order to provide healthcare services.

In the guidelines issued concerning COPD, early detection activities are important due to the expectation of a higher number of undiagnosed patients [6, 14]. Several different settings and ways of handling early detection have previously been investigated, that is, “walk-in” initiatives [18] as well as GP initiatives [10, 19]. Since 2007, municipalities in Denmark had by legislation the responsibility for disease prevention, health promotion, and rehabilitation of citizens with chronic diseases. A governmental prevention committee suggested in terms of disease prevention and health promotion municipalities to be one potential arena for early detection of COPD, in addition to the GP setting [20]. A pilot study in one Danish municipality has indicated that a high rate of detection of COPD is feasible in the municipality setting extending the possibilities to detect COPD patients early in order to prevent disease progression [21]. A number of municipalities have since this pilot study shown an interest in early diagnosis of COPD and have built this into their current tasks and obligations, also in accordance with the new guidelines on case finding of COPD issued by the Danish National Board of Health [14].

The purpose of the present study was therefore to provide an overview and status on the guideline-based general concept of municipality early detection and case finding of pulmonary obstruction among citizens under suspicion of having undiagnosed COPD with data from eight Danish municipalities.

## 2. Materials and Methods

### 2.1. Study Design

A case finding study was conducted in eight municipalities (Esbjerg, Frederikshavn, Billund, Jammerbugt, Struer, Gribskov, Solrød, and Vordingborg) covering in total 8% (430,000 citizens) of the Danish population and with a geographical spread throughout Denmark. The study was designed to follow usual procedure adapted in the different municipalities for early detection of pulmonary obstruction (COPD). This could for example be a “walk-in” setting in the municipality healthcare center, case finding sessions for patients (without the diagnosis of COPD) already being treated or rehabilitated in the municipality healthcare center, and sessions at shopping centers, workplaces, and so forth. An easy to use Internet database was developed in cooperation with and hosted externally by the Danish Technical University. The database consisted of indicators and variables described in the current recommendation for detection of COPD issued by the Danish National Board of Health [8]. All citizens were given written and oral informed consent before initiating the case finding procedures. The study was approved by the Danish Data Protection Agency. The study protocol was also submitted to the local ethics committee and the Danish Medicines Agency for approval. However, being a noninterventional study, both authorities judged that the study did not require their approval.

### 2.2. Included Citizens and Case Finding Procedure

Citizens that fulfilled the recommendations for case finding for COPD issued by the Danish National Board of Health, that is, age ≥35 years, smokers/ex-smokers and/or relevant occupational exposure, and at least one respiratory symptom (dyspnea, cough, wheeze, sputum, and recurrent lower respiratory tract infections), were offered spirometry to investigate indications of obstruction. Citizens with a diagnosis of COPD were excluded. Based on daily practice the spirometry conducted was in accordance with the Danish Respiratory Society guidelines, and the municipality healthcare personnel were educated in conducting reproducible and conclusive spirometry. Spirometers were calibrated before initiation of the study in accordance with the provider recommendation. The procedure included at least three-times forced exhalation under supervision and at least two successful measurements of forced expiratory volume in the first second (FEV_1_) together with forced vital capacity (FVC). Two of the spirometry examinations had to differ by less than 5 percent to be considered valid.

An FEV_1_/FVC ratio of <0.70 was defined as pulmonary obstruction according to the Global Initiative of Chronic Obstructive Lung Disease (GOLD) [1]. An initial calculation of a possible COPD staging was done based on the FEV_1_ value following GOLD guidelines: mild: FEV_1_ ≥80%, moderate: FEV_1_ <80% to ≥50%, severe: <50% to ≥30%, and very severe: <30% of expected value.

Citizens were asked about smoking status and presence of respiratory symptoms and questions concerning their dyspnea using the Medical Research Counsel scale (MRC). After the spirometry active smokers were asked if they had participated in smoking cessation initiatives previously and if advice had been accompanied by medical treatment and if smoking abstinence had been achieved. Finally, all smokers were asked about their interest in future smoking cessation attempts. No information as to comorbidities, besides the respiratory symptoms, was accessed at the time of the examination in the municipalities.

All participants with indication of pulmonary obstruction were advised to visit their general practitioner for further spirometry testing, including reversibility testing for a more specific diagnosis [21], a subsequent treatment, and other indicated measures. Smoking abstinence was self-reported point prevalence.

### 2.3. Statistical Analyses

The data were analyzed using IBM SPSS Statistics version 22.0 (IBM Corporation, Armonk, New York). For comparisons between subjects with evidence of airflow obstruction and those without, between men and women, and between smokers and nonsmokers, the Chi-square test and *t*-test were used depending on the data analyzed and the scale level of these data. A significance level of 5% was chosen.

## 3. Results

1,499 citizens in the eight municipalities were examined (53.6% male) with a mean age of 57.2 (range 36–92). At the time of the examination 96% of the citizens were smokers or ex-smokers, 7% were at risk because of occupational exposure, and 5% had other risk factors.  Table 1 shows the descriptive parameters as well as spirometry results.

**Figure 1:**
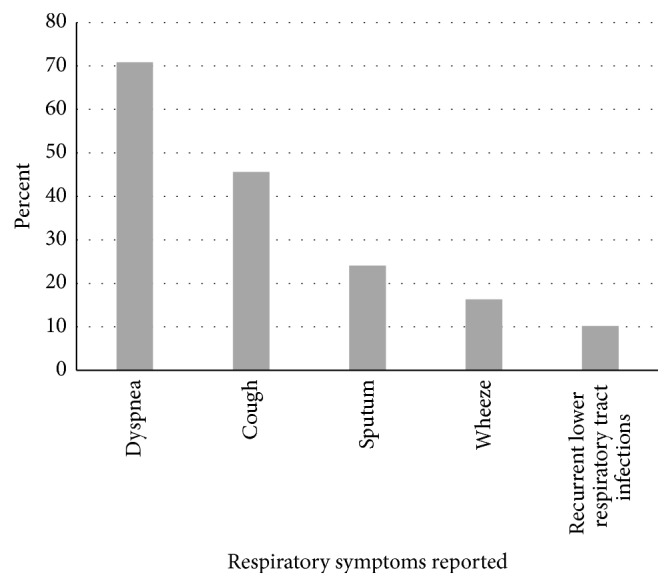
Respiratory symptoms in the citizens examined (*N* = 1,499).

Figure 1 shows the different respiratory symptoms at the time of the examination.

The MRC average score was 1.55 (SD 0.76) (Table 2).

456 out of the 1,499 citizens examined (30.4%: females 26.1%, males 35.5%) were found to have an indication for pulmonary obstruction based on the spirometry examination. Across the municipalities, the rate of citizens with indication of pulmonary obstruction following spirometry varied between 22% and 44%. Obstruction among citizens that were current smokers and ex-smokers and never smoked was 32.8%, 29.1%, and 18.8%, respectively.

Based on the spirometry result, indications of COPD staging split into mild, moderate, severe, and very severe were, respectively, 10.4%, 15.5%, 3.7%, and 0.8% among the total number of citizens examined or split into 34% mild, 50% moderate, 12% severe, and 3% very severe cases of COPD among those with an indication of obstruction. The dyspnea stages of the patient groups were analyzed to the different groups of pulmonary obstruction versus the mean MRC score showing a significant increase in the MRC score with COPD severity stage (Table 2).

For those citizens with a result indicating pulmonary obstruction the municipalities advised the citizen to approach their own GP for further clinical evaluation and diagnosis.

There were 670 current smokers corresponding to 44.8% of all citizens examined (females 47.1%, males 42.1%, NS). Current and ex-smokers (96.8% of all citizens) had smoked an average of 16 cigarettes a day (SD 9.8) and a total of 28 pack years (current smokers: 31 pack years). An insignificant part of the citizens had smoked other tobacco types, that is, pipe and cigar. There were no differences between males and females in terms of their wish to participate in future smoking cession initiatives. Severity of obstruction between ex-smokers and current smokers showed that there was a trend—although insignificant—towards more ex-smokers in the group with mild disease (53.8% of the ex-smokers versus 45.5% of the current smokers) than in citizens with indication of very severe obstruction (41.7% of the ex-smokers versus 58.3% of the current smokers).

Nearly three-fourths of all current smokers had previously tried to quit smoking (Table 3). However, still more than 57% were interested in future smoking cessation attempts right away after spirometry (Now) or for the majority planning this after some time (Planned).

## 4. Discussion

The present study confirmed that it is possible and effective to perform case finding of pulmonary obstruction in the municipality settings in Denmark. Many citizens with undiagnosed disease (456 out of 1,499 citizens (30.4%)) of those fulfilling the national criteria for case finding had indication for pulmonary obstruction. They were advised to attend their GP for further examination to confirm diagnosis of COPD. Evidence from a pilot study in one municipality showed that among those citizens found with indication of pulmonary obstruction in the municipality 85% were diagnosed with COPD at the GP afterwards [21]. Extrapolated to the eight municipalities in the present study this corresponds to 388 citizens potentially being diagnosed with COPD.

Several other studies have demonstrated that it is effective to perform early detection at GP's, walk-in study activities, and so forth [10, 18, 19]. MRC could be an initial measurement in the early detection of COPD, as the dyspnea burden is the most prominent problem for the patient. Subjects found by early detection with spirometry and later beta2 agonist reversibility testing have previously been analyzed to assess reversibility to see if the algorithm issued for COPD case finding is valid [22]. The conclusion was that reversibility is important but should not stand in the way for essential early detection of COPD confirming the procedure in the present study [22].

Concurrently the study opens a major differential diagnosis: asthma or COPD: In recent years it has been debated, how many patients in this population do have the Asthma-COPD Overlap Syndrome (ACOS). It is estimated that between 15% and 25% of obstructive patients suffer from ACOS. Guidelines for early detection and treatment by both GOLD and GINA are currently being developed. We have, however, not been able to address ACOS, but other previous Danish studies support the fact that a clear majority have COPD [10, 19].

The share of current smokers was surprisingly high in the population examined in the present study (45%) compared to the overall smoking prevalence in Denmark (17%) [11]. High smoking prevalence, heavy smokers, and a long history of smoking indicate a high prevalence of nicotine addiction. In the study population this is potentially higher than the share of nicotine dependence of 60% expected among all current smokers in Denmark [23]. A high degree of nicotine dependence is supported by the high burden of pulmonary symptoms found in the study among citizens still smoking. For example, 70% reported severe dyspnea. Current smokers have a higher prevalence of pulmonary obstruction compared to ex-smokers and never smokers. However, smoking cessation is obvious for all citizens smoking to avoid smoking-related diseases, as well as progression of the COPD disease.

Besides undertaking case finding activities, municipalities can secure that smoking citizens are offered smoking cessation. Forty percent of those who were smoking in the pilot study continued to be abstinent three months later, due to the fact that there is a motivation to quit smoking among those who were detected [21]. Following the recommendations given by the Danish National Board of Health for all smokers [14, 23, 24], the most effective smoking cessation therapy offered to smokers with or without COPD is combinations therapy (smoking cessation advice + smoking cessation medicine). For patients with COPD who continue smoking the recommendations emphasize that smoking cessation is the first intervention towards progression of COPD [8].

The results of the present study indicate that the municipalities are an additional arena for early detection and case finding of pulmonary obstruction and COPD of their citizens besides GPs and hospitals. This follows the recommendations at the national level to have more undiagnosed COPD patients detected and diagnosed leading to less disease progression and severity of the COPD disease in Denmark [14]. Covering both large and smaller municipalities the result of the study is regarded as fairly representative for the rest of the Danish municipalities. Extrapolating the result to all 98 municipalities assuming the same case finding result overall will then lead to early identification of around 6,500 citizens with pulmonary obstruction and around 5,530 ending up with confirmed COPD. Daily routine case finding over longer time periods would increase these figures and show that early detection of COPD is worthwhile.

## Figures and Tables

**Table 1:** Descriptive statistics and spirometry results (*N* = 1,499).

	Mean	SD
Height (cm)	171.78	8.79
Weight (kg)	77.96	15.95
BMI	26.32	4.51
FEV1 (liter)	2.64	0.81
FVC (liter)	3.61	0.97
FEV1/FVC ratio	73.54	22.84
FEV1% of expected	87.00	19.66

**Table 2:** Medical Research Council (MRC) average score in estimated severity in groups of COPD^**∗**^ (*N* = 1,499).

Estimated severity of COPD	Mean	SD
No obstruction	1.47	0.68
Mild COPD	1.39	0.66
Moderate COPD	1.76	0.84
Severe COPD	2.25	1.09
Very severe COPD	3.00	1.35

^*∗*^Chi-square 88.8; *P* < 0.001.

**Table 3:** Current smokers: smoking cessation (*N* = 670).

Interest in future smoking cessation?	“Not interested”	43%
	“Planned”	44%
	“Now”	13%

Previously tried smoking cessation?	“Yes”	71%
	“No”	29%

Former medical treatment to assist in smoking cessation?	“None”	53%
	“Nicotine replacement”	22%
	“Prescription medication”	9%
